# Drug targeting of aminoacyl-tRNA synthetases in *Anopheles* species and *Aedes aegypti* that cause malaria and dengue

**DOI:** 10.1186/s13071-021-05106-5

**Published:** 2021-12-11

**Authors:** Soumyananda Chakraborti, Jyoti Chhibber-Goel, Amit Sharma

**Affiliations:** 1 Molecular Medicine Group, National Institute of Malaria Research, New Delhi, India; 2 Molecular Medicine, International Centre for Genetic Engineering and Biotechnology, New Delhi, India

**Keywords:** Aminoacyl-tRNA synthetases, Genomics, *Anopheles* spp., *Aedes* spp., Drug discovery

## Abstract

**Background:**

Mosquito-borne diseases have a devastating impact on human civilization. A few species of *Anopheles* mosquitoes are responsible for malaria transmission, and while there has been a reduction in malaria-related deaths worldwide, growing insecticide resistance is a cause for concern. *Aedes* mosquitoes are known vectors of viral infections, including dengue, yellow fever, chikungunya, and Zika. Aminoacyl-tRNA synthetases (aaRSs) are key players in protein synthesis and are potent anti-infective drug targets. The structure–function activity relationship of aaRSs in mosquitoes (in particular, *Anopheles* and *Aedes* spp.) remains unexplored.

**Methods:**

We employed computational techniques to identify aaRSs from five different mosquito species (*Anopheles culicifacies*, *Anopheles stephensi*, *Anopheles gambiae*, *Anopheles minimus*, and *Aedes aegypti*). The VectorBase database (https://vectorbase.org/vectorbase/app) and web-based tools were utilized to predict the subcellular localizations (TargetP-2.0, UniProt, DeepLoc-1.0), physicochemical characteristics (ProtParam), and domain arrangements (PfAM, InterPro) of the aaRSs. Structural models for prolyl (PRS)-, and phenylalanyl (FRS)-tRNA synthetases—were generated using the I-TASSER and Phyre protein modeling servers.

**Results:**

Among the vector species, a total of 37 (*An. gambiae*), 37 (*An. culicifacies*), 37 (*An. stephensi*), 37 (*An. minimus*), and 35 (*Ae. aegypti*) different aaRSs were characterized within their respective mosquito genomes. Sequence identity amongst the aaRSs from the four *Anopheles* spp. was > 80% and in *Ae. aegypti* was > 50%.

**Conclusions:**

Structural analysis of two important aminoacyl-tRNA synthetases [prolyl (PRS) and phenylanalyl (FRS)] of *Anopheles* spp. suggests structural and sequence similarity with potential antimalarial inhibitor [halofuginone (HF) and bicyclic azetidine (BRD1369)] binding sites. This suggests the potential for repurposing of these inhibitors against the studied *Anopheles* spp. and *Ae. aegypti*. 
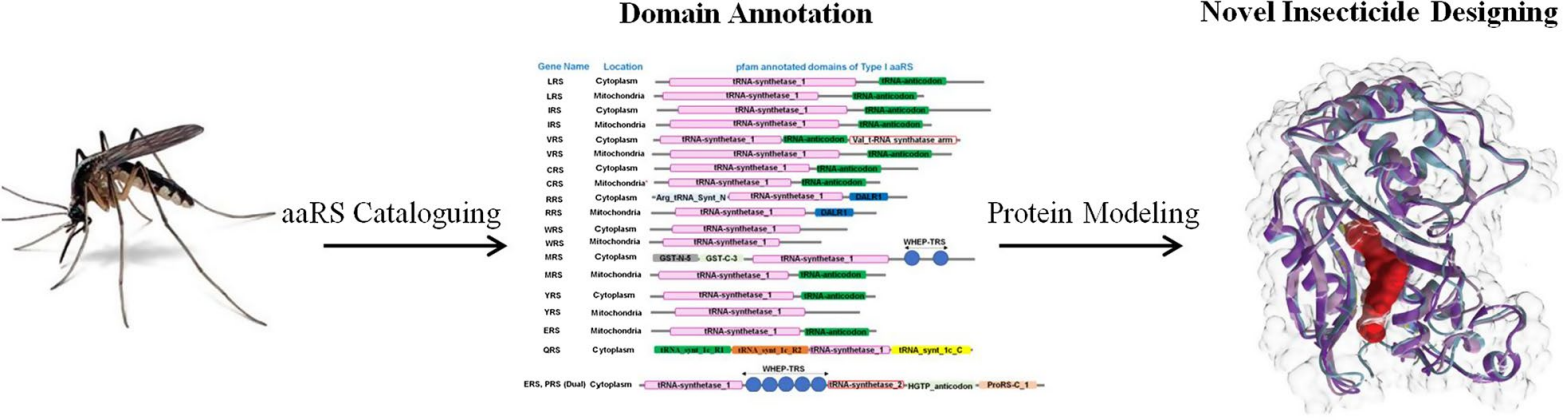

**Supplementary Information:**

The online version contains supplementary material available at 10.1186/s13071-021-05106-5.

Each year approximately 700,000 deaths (~ 17% of total infectious disease deaths worldwide) are attributed to mosquito-borne diseases such as chikungunya, dengue, malaria, West Nile virus, and Zika [1–3]. Mosquitoes transmit diseases by harboring the disease-causing parasites or viruses that are transmitted to the host during blood-feeding [3, 4]. Increased insecticide resistance makes efforts to control mosquitoes challenging [5–7]. Worldwide there are over 3500 mosquito species [8]. Among the different species of mosquitoes, *Aedes aegypti* transmits viruses (e.g., dengue, yellow fever, chikungunya, and Zika), whereas *Anopheles* spp. transmit the malaria-causing parasites of *Plasmodium* spp. [9]. Regarding malaria, the three primary *Anopheles* vectors in India are (i) *An. culicifacies* (rural areas), which is responsible for the majority of malaria cases in India; (ii) *An. stephensi* (urban areas); and (iii) *An. minimus* (northeastern region of India) [10, 11]. *Anopheles gambiae* is highly effective in spreading malaria and is mainly localized to Africa. In addition to *Plasmodium falciparum*, *An. gambiae* hosts and transmits the filarial worm *Wuchereria bancrofti* that causes lymphatic filariasis [12]. With recent advancements in sequencing technologies, the full genomes of several mosquito species have been decoded and new protein targets identified. These findings hold promise for the discovery and development of novel insecticides [13].

Aminoacyl-tRNA synthetases (aaRSs) are also known as tRNA ligases, and they universally drive the protein translation process [14, 15]. The aminoacylation reaction catalyzed by aaRSs provides an opportunity for the development of protein translation inhibitors [16–20]. The main function of aaRSs is to append an amino acid to the respective tRNAs in an adenosine 5′-triphosphate (ATP)-dependent manner. First, ATP activates an amino acid to form an intermediate molecule known as “aminoacyl-adenylate.” In the next step, the intermediate is attached to the cognate tRNA molecule through covalent bond formation and the reaction is completed with the release of adenosine 5′-monophosphate (AMP) [21]. The aaRSs are multi-domain proteins with (i) a conserved catalytic domain (responsible for tRNA and amino acid ligation events), (ii) an anticodon binding domain (ABD) (responsible for binding of the anticodon region of the tRNA), and other additional domains for (iii) RNA binding and (iv) editing activity, or (v) C-terminal zinc-binding-like domain. The editing domain is responsible for removing incorrectly charged tRNA. In nature, there are 20 amino acids, and in general, for each amino acid there is at least one aaRS present. These 20 aaRSs can be broadly classified into either class I or class II. Class I and class II enzyme annotation is based on two factors: (i) possession of conserved structural motifs and (ii) their mode of substrate binding. Typical characteristics of class I enzymes are possession of a Rossmann fold, which is composed of two highly conserved motifs, KMSKS and HIGH. Compared to the class I type, class II aaRSs contain three conserved motifs in their domain and carry a unique fold composed of antiparallel beta strands [22]. The aaRSs show high sequence diversity, with structural differences and domain arrangements across organisms; however, aaRS catalytic domains are more conserved across species. Further, noncanonical functions of aaRSs include RNA splicing, transcription regulation, signal processing, immune responses, and apoptosis [23, 24]. For instance, the *P. falciparum* tyrosyl-tRNA synthetase (*Pf*YRS) possesses cytokine-like activity [25]. The aaRSs are mainly located in the cytoplasm and the mitochondria for protein synthesis [26]. Recently, aaRSs have also emerged as a potential drug target for several eukaryotic pathogens (*Leishmania*, *Plasmodium*, and *Toxoplasma*) via multi-site targeting [16–18, 25, 27–35].

In the present study, computational tools were employed to characterize aaRSs present in the genomes of *Anopheles* (*An. gambiae*, *An. culicifacies*, *An. stephensi*, and *An. minimus*) and *Ae. aegypti*. All the different aaRSs present in the five studied mosquito species were annotated. Our analysis identified a total of 37 (*An. gambiae*), 37 (*An. culicifacies*), 37 (*An. stephensi*), 37 (*An. minimus*), and 35 (*Ae. aegypti*) different aaRSs in these respective genomes. We also investigated individual aaRS sequences in detail and predicted their isoelectric point (pI) and potential subcellular localizations. Furthermore, we determined the domain arrangements of all different aaRS spread across five different mosquito species and generated structural models of several important druggable targets. Overall, this study lays the groundwork for the development of next-generation insecticides against the *Anopheles* spp. and *Ae. aegypti* aaRSs.

Protein sequences of the aaRSs from the five mosquitoes (*An. culicifacies*, *An. stephensi*, *An. minimus*, *An. gambiae*, *Ae. aegypti*) were retrieved from the VectorBase database (https://vectorbase.org/vectorbase). The VectorBase (https://vectorbase.org/vectorbase) annotated protein sequences were further validated by comparing the sequences from the NCBI (https://www.ncbi.nlm.nih.gov/) and UniProt (https://www.uniprot.org/) databases. ProtoParam (https://web.expasy.org/protparam/) was used to characterize individual aaRS isoelectric points. VectorBase (https://vectorbase.org/vectorbase) was initially used to extract the protein sequence of aaRSs from *An. gambiae*, and this was used as a reference for genome annotation of the other mosquito species studied here via Protein BLAST. The aaRS sequences from *An. culicifacies, An. stephensi, An. minimus*, and *Ae. aegypti* were further screened against each other in various databases, including NCBI (https://www.ncbi.nlm.nih.gov/) and UniProt (https://www.uniprot.org/), for further validation.

Subdomains of individual aaRSs were determined using the online web servers Pfam (http://pfam.xfam.org/) and InterPro (https://www.ebi.ac.uk/interpro/). The same databases were also used to acquire information about domain function. The aaRS signal sequence/peptide and subcellular localization were predicted using the online web servers TargetP-2.0 (http://www.cbs.dtu.dk/services/TargetP/), SignalP 5.0 (http://www.cbs.dtu.dk/services/SignalP/), and DeepLoc-1.0 (http://www.cbs.dtu.dk/services/DeepLoc/). Multiple sequence alignment (MSA) of the drug binding site was performed utilizing MUSCLE (https://www.ebi.ac.uk/Tools/msa/muscle/). MSA for phylogenetic analysis was carried out using Clustal Omega (https://www.ebi.ac.uk/Tools/msa/clustalo/).

Homology modeling of lysyl (KRS)-, prolyl (PRS)-, and phenylalanyl-tRNA (FRS) synthetases was carried out using two different web-based servers: Phyre (http://www.sbg.bio.ic.ac.uk/~phyre2/html/page.cgi?id=index) and I-TASSER (https://zhanglab.ccmb.med.umich.edu/I-TASSER/server). The quality of the protein models was assessed using SAVES v6.0 (https://saves.mbi.ucla.edu/). Protein structure visualization was done using PyMOL (https://pymol.org/2/) and Chimera (https://www.cgl.ucsf.edu/chimera/download.html).

Our analyses identified 37 aaRSs enzymes in all four *Anopheles* spp. (*An. culicifacies*, *An. stephensi*, *An. minimus*, *An. gambiae*) and 35 in *Ae. aegypti*, including the bifunctional aaRSs—glutamyl-prolyl-tRNA synthetase (EPRS) (Fig. 1, Additional file 1: Table S1) [36]. *Aedes aegypti* carries only one copy of histidyl-(HRS) and lysyl-tRNA synthetase (KRS), whereas the four *Anopheles* spp. carry two copies each of KRS and HRS (Additional file 1: Table S1). All five mosquito species (four *Anopheles* spp. and *Ae. aegypti*) possess two copies of each aaRS, except for glycyl-(GRS), threonyl-(TRS), and glutaminyl-(QRS) tRNA synthetases, with one gene copy; and FRS, which has three copies, with two coding for the one FRS-alpha-like, one for FRS-alpha, and one for the FRS-beta subunit.

**Fig. 1:**
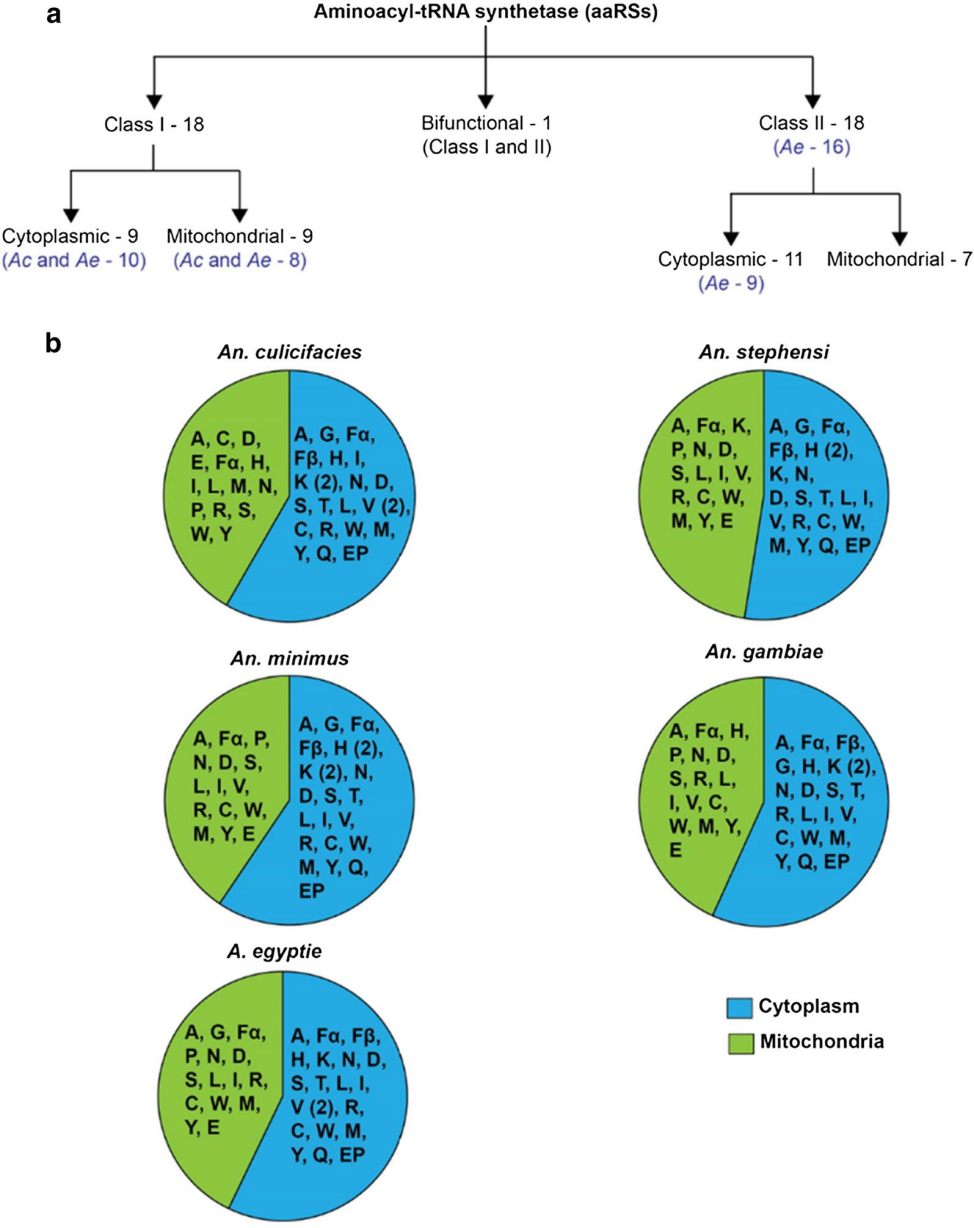
The distribution of aminoacyl-tRNA synthetases (aaRSs) in the studied mosquito species. aaRSs from *Anopheles* (*An. culicifacies*, *An. stephensi*, *An. minimus*, and *An. gambiae*) and *Ae. aegypti* were characterized based on topologies and subcellular location. **a** Class I and II annotation was performed based on the topologies of the central catalytic domain (CCD) and the mode of substrate binding. The number of aaRSs annotated for each studied species and their localization for class I, class II, and bifunctional aaRSs is shown, with exceptions listed in brackets. For class I aaRSs, *An. gambiae*, *An. stephensii*, and *An. minimus* carry nine cytoplasmic and nine mitochondrial aaRSs, whereas *An. culicifacies* and *Ae. aegypti* have 10 cytoplasmic and eight mitochondrial aaRSs. **b** The tentative distribution of the predicted subcellular localizations (cytoplasmic and mitochondrial) for the studied *An. culicifacies*, *An. stephensi*, *An. minimus*, *An. gambiae*, and *Ae. aegypti* is shown

The aaRSs are generally classified into two groups, class I and class II, based on the conserved topology of the synthetase core domain. We observed an equal number of proteins belonging to class I and II families (18 each), except for *Ae. aegypti*, where 18 class I and 16 class II type aaRSs were found (Fig. 1 and Additional file 1: Table S1). Additionally, a single protein copy of the bifunctional EPRS was present in all the studied mosquito species. The subcellular localization of the annotated aaRSs from the mosquito species was predicted using online servers. *Anopheles gambiae* and *An. stephensi* have 21 aaRSs localized to the cytoplasm and 16 to the mitochondria (Fig. 1 and Additional file 1: Table S1), whereas in *An. minimus* and *An. culicifacies*, out of a total of 37 aaRS enzymes, 22 are localized to the cytoplasm and 15 to the mitochondria. In *Ae. aegypti*, out of 35 aaRSs, 20 are predicted to localize to the cytoplasm and 15 to the mitochondria (Fig. 1). Consistent with other species such as *Babesia* spp., *Plasmodium* spp., and *Homo sapiens*, a larger number of aaRSs are found in the cytoplasm compared to the mitochondria [34, 37]. Generally, mitochondrial-targeting peptide is present at the N- or C-terminus or at the internal site of the protein [26]. It is worth noting that protein/tRNA migration to the mitochondria has been reported in the absence of signal peptides [38]. For instance, the charged tRNAs of the aaRSs are missing within the mitochondrial compartment, and they are imported from the cytoplasm in protozoa of the genera in *Leishmania*, *Trypanosoma*, *Plasmodium*, and *Toxoplasma* [39–43].

The aaRSs consist of multiple domains, with a core synthetase domain and several other auxiliary domains for RNA binding, editing, and oligomerization, some of which have been targeted for drug discovery [18]. Our analysis of the five mosquito species suggests the presence of a core catalytic domain and several additional domains for all studied aaRSs. For instance, the DALR [aspartate (D), alanine (A), leucine (L), arginine (R)] is an anticodon binding domain, which largely consists of an α-helical structure and was found in arginyl tRNA synthetase (RRS) of all mosquito species (Fig. 2 and Additional file 1: Table S1). The DHHA [aspartate (D), histidine (H), histidine (H), alanine (A)] domain is unique to the cytoplasmic variant of the (alanyl tRNA synthatase) ARS enzyme [44]. Furthermore, Pfam predicted the presence of the glutathione S-transferases (GST)-like domain in the cytoplasmic version of methionyl-tRNA synthetase (MRS). Two GST subdomains at the N- and C-terminus were also present within MRS from all the studied mosquito species (Additional file 1: Table S1). GST or GST-homology domains play a crucial role in aaRS complex formation with multifunctional factors such as p18, p38, and p43 [45, 46]. The second additional domain (SAD), WHEP-TRS (also known as helix-turn helix domain or Wh-T), TGS [TRS (threonyl-tRNA synthetase), GTPase, and SpoT], and the HGTP anticodon domain were also present in several of the studied aaRSs (Fig. 2). For example, the Wh-T domain was mainly detected in bifunctional EPRS of all five mosquito species, and our analysis further revealed the presence of multiple copies of the same (Wh-T) domain within the EPRS structure. Other than EPRS, the Wh-T domain was also found in the cytoplasmic version of MRS in all *Anopheles* and *Aedes* mosquitoes (Additional file 1: Table S1). The Wh-T domain was detected in the GRS enzyme of all mosquitoes examined here as well. The same domain was also found in the cytoplasmic variant of HRS in all *Anopheles* and *Aedes* species, the only exception being *An. culicifacies* HRS. These domains are well known for their interaction with the GAIT (interferon-gamma-activated inhibitor of translation) complex [47]. Additionally, these domains are involved in tRNA binding to the aaRS [48]. Intriguingly, one of the three FRS genes (cytoplasmic variants of FRS-alpha) encodes multiple DNA binding domains (DBD) [49] in all four *Anopheles* spp. and *Ae. aegypti* analyzed here (Fig. 2). Our analysis detected the presence of a secondary associated domain (tRNA_SAD) in all ARS and TRS examined (Fig. 2 and Additional file 1: Table S1). This domain generally contains a highly conserved HxxxH motif that is frequently present in metal-dependent hydrolases [50]. In addition to the SAD domain, TRS possesses a TGS domain that is located at the N-terminus of the protein [44], and the TGS domain was found to be present in all mosquito species. Additionally, a comparison of class I and class II aaRSs clearly showed the presence of a higher number of subdomains in class II enzymes.

**Fig. 2:**
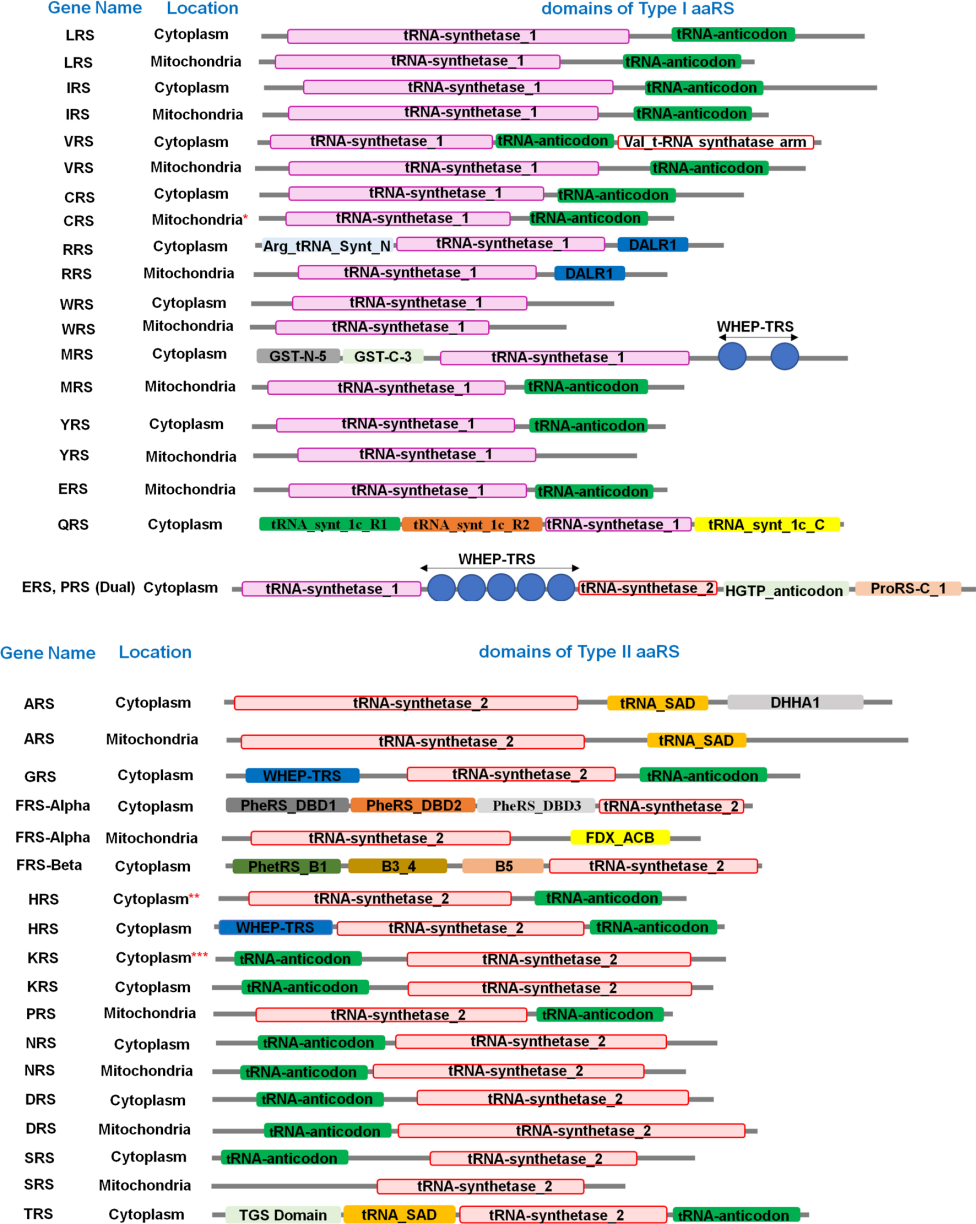
Pfam (http://pfam.xfam.org/) and UniProt (https://www.uniprot.org/) assigned common domain features of all catalogued aaRS enzymes from four different *Anopheles* species and *Aedes* spp. Detailed domain arrangement of class I and class II type aaRSs across different mosquito species. Protein sequences of the aaRS gene (both class I and class II) were retrieved from VectorBase (https://vectorbase.org/). For CRS, HRS, and KRS, cytoplasmic and mitochondrial variants are not uniformly distributed across different species analyzed here

Analysis of the physicochemical properties for all aaRSs in this study (isoelectric point [pI]) showed that in general, the core synthetase domain of class II aaRSs is more acidic in nature and carries a large number of negatively charged residues (on the surface) compared to the class I synthetase (Additional file 1: Table S2). However, the length of the core synthetase domain belonging to class I aaRSs was found to be greater than that of the class II family of the aaRS enzymes (Additional file 1: Table S2).

Further analysis of the sequence identity between the core synthetase domain of the annotated aaRSs from *An. culicifacies* and *An. gambiae* in comparison with *H. sapiens* is shown in Additional file 1: Table S3. The core synthetase domain of the aaRSs from *An. culicifacies* and *An. gambiae* shared ~ 90–95% sequence identity (data not shown), whereas these two species shared sequence identity of ~ 39–82% with aaRSs from *H. sapiens* (Additional file 1: Table S3). In the case of cytoplasm variants, the maximum sequence identity (~ 82%) was observed for seryl t-RNA synthatase (SRS) and the minimum sequence identity was found in valyl-tRNA synthetase (VRS) from *An. culicifacies* (~ 55%). Our analysis showed that mitochondrial aaRSs have poor sequence identity in comparison with their cytoplasmic counterparts when compared to the human homolog (Additional file 1: Table S3). The moderate difference in sequence identity between *Anopheles* and human aaRSs shows promise for the design of insecticides selectively targeting mosquitoes.

Three of the aaRSs—lysyl (KRS), prolyl (PRS), and phenylanalyl (FRS)—are advanced targets for drug discovery against malaria parasites and have been structurally and functionally validated against either *P. falciparum* or *Plasmodium vivax.* The importance of structure-based targeting of orthologous pathogen proteins (STOPP) to accelerate the discovery of novel drugs via the assessment of sequence conservation within the active site residues in aaRSs has been widely explored [28]. The sequence similarity between the aaRSs from the four *Anopheles* spp. and *Ae. aegypti* was of a moderate range (i.e., 40–80%). Thus, in order to explore the possibility of exploiting three of the advanced aaRS antimalarial drug targets KRS, PRS, and FRS in *Anopheles* and *Ae. aegypti*, we determined the sequence similarity between KRS, PRS, and FRS of different mosquito species compared to *P. falciparum* and *P. vivax*. Our analysis showed partial sequence conservation amongst the five mosquito species with both *P. falciparum* (KRS ~ 32–55%, PRS ~ 19–29% range, FRS-*alpha* ~ 42–44% range) and *P. vivax* (KRS ~ 31–54% range, PRS ~ 19–29% range, FRS-*alpha* ~ 40–42% range), with high active-site conservation that was further explored in our analysis. Hence, as proof of concept, we built three-dimensional models of KRS, PRS, and FRS enzymes from one of the five mosquito species (i.e., *An. culicifacies*), as previously described in the methods section. *Anopheles culicifacies* was chosen as it is the most prevalent and the primary malaria-causing vector in India [11]. Analysis of the active site residues of these three (*pf*KRS, *Hs*KRS, and *Ac*KRS) aaRSs showed poor active site residue conservation in KRS (data not shown). We subsequently analyzed the halofuginone active site in PRS and bicyclic azetidine compound active site in FRS.

*Plasmodium falciparum* PRS (*Pf*PRS) has been studied as a drug target of halofuginone (HF) [32, 51, 52]. Sequence and structural comparison was performed between the *Pf*PRS (PDB: 4YDQ), *Hs*PRS (PDB: 4K88), and *Ac*PRS (modeled three-dimensional structure) (Fig. 3a). The analyses revealed the HF active site to be partly conserved (Fig. 3b) [32]. Among the active sites, the bulkier residues in *Pf*PRS and *Hs*PRS were replaced with the smaller residues in *Ac*PRS–for example, Tyr with Thr, Thr with Ser, Phe with Tyr (Fig. 3c). In addition to *Pf*PRS, an inhibitor bound structure for the *P. vivax* FRS (*Pv*FRS) was also analyzed (*Pf*FRS structure is not available). Structural analysis of the *Pv*FRS (PDB: 7BY6), *Hs*FRS (PDB: 3L4G), and the *Ac*FRS was performed to characterize the active site residues of the BRD1389 bound inhibitor of FRS (Fig. 4a) [35]. Our analysis revealed that the active site within the *Pv*FRS**,** *Hs*FRS, and the modeled *Ac*FRS is highly conserved (Fig. 4b). A comparison with the *Hs*FRS^cyto^ revealed that the *Pv*FRS^cyto^ occupies a ligand-induced fit model based on open conformation of the loop formed by the ATP binding pocket residues numbered 443–453 to accommodate the methoxymethyl group. On the other hand, closed conformation of residues is observed in the auxiliary pocket [35, 36]. Further, the key BRD1389 interacting residues as shown in Fig. 4c within the active site were highly conserved in comparison to the PvFRS.

**Fig. 3:**
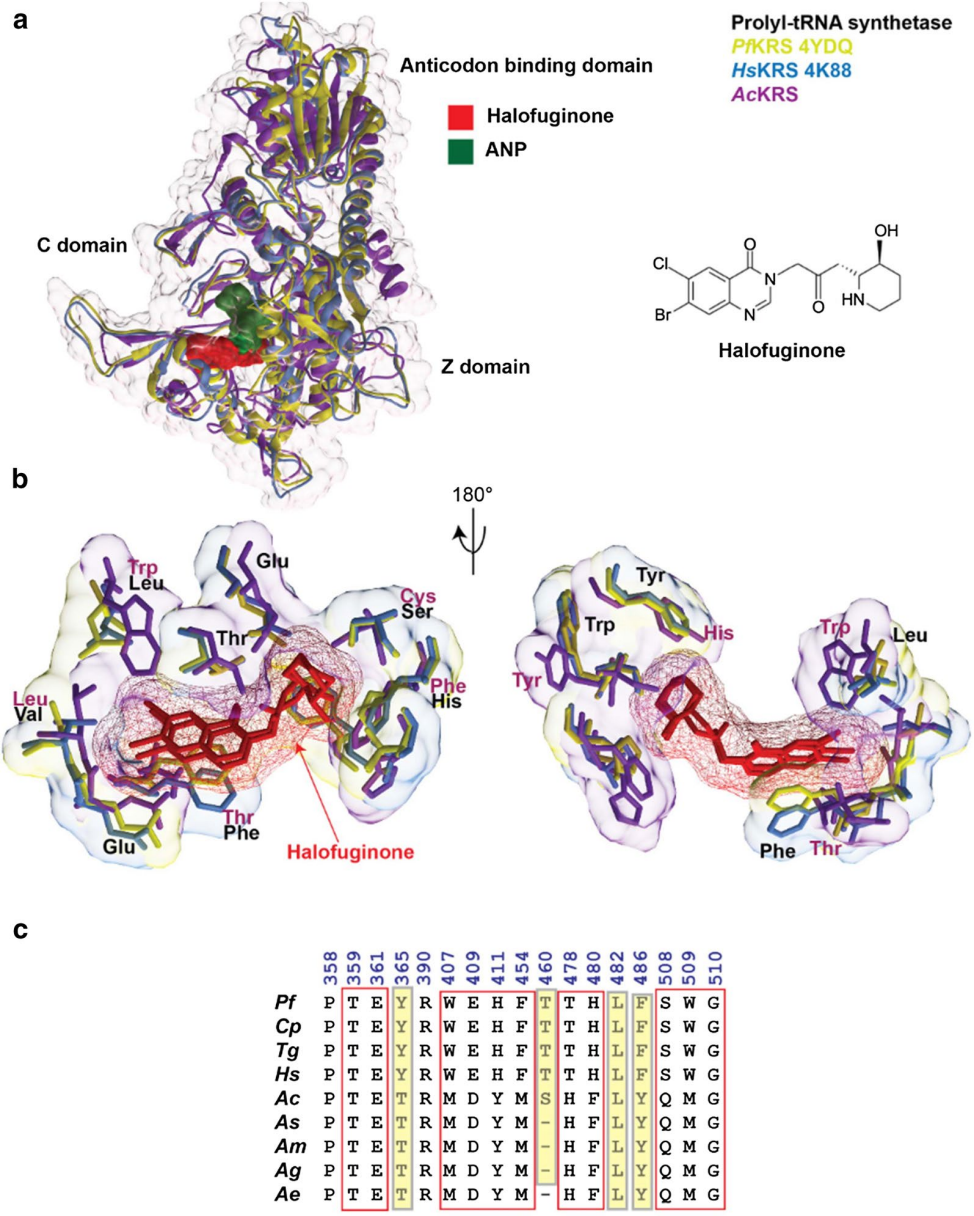
Halofuginone binding site in prolyl-tRNA synthetase from *An. culicifacies* in comparison with *P. falciparum* and *H. sapiens.* **a** Catalytic (C domain), anticodon binding and the C-terminal zinc-binding-like (Z domain) domains are marked on the three-dimensional crystal structure of holo-prolyl-tRNA synthetase from *Hs*PRS (PDB: 4K88) (blue), *Pf*PRS (PDB: 4YDQ3) (yellow), and *Ac*PRS (built structure model, this study) (purple) are shown. Halofuginone (HF) is shown in red and phosphoamniophosphonic acid-adenylate ester (ANP) in green. The chemical structure of HF (halofuginone) is also shown (marked in red) in the same figure. **b** Structural superposition of the three-dimensional crystal structure with the key HF interacting residues is shown for *P. falciparum*, *H. sapiens*, and *An. culicifacies*. **c** Multiple sequence alignment of the HF binding site residues (red box) and the important secondary shell residues as determined from the known three-dimensional crystal structures across species is shown. *Pf*: *P. falciparum*; *Cp*: *Cryptosporidium parvum*; *Tg*: *Toxoplasma gondii*; *Hs*: *H. sapiens*; *Ac*: *An. culicifacies*; *As*; *An. stephensi*; *Am*: *An. minimus*; *Ag*: *An. gambiae*; *Ae*: *Ae. aegypti*

**Fig. 4:**
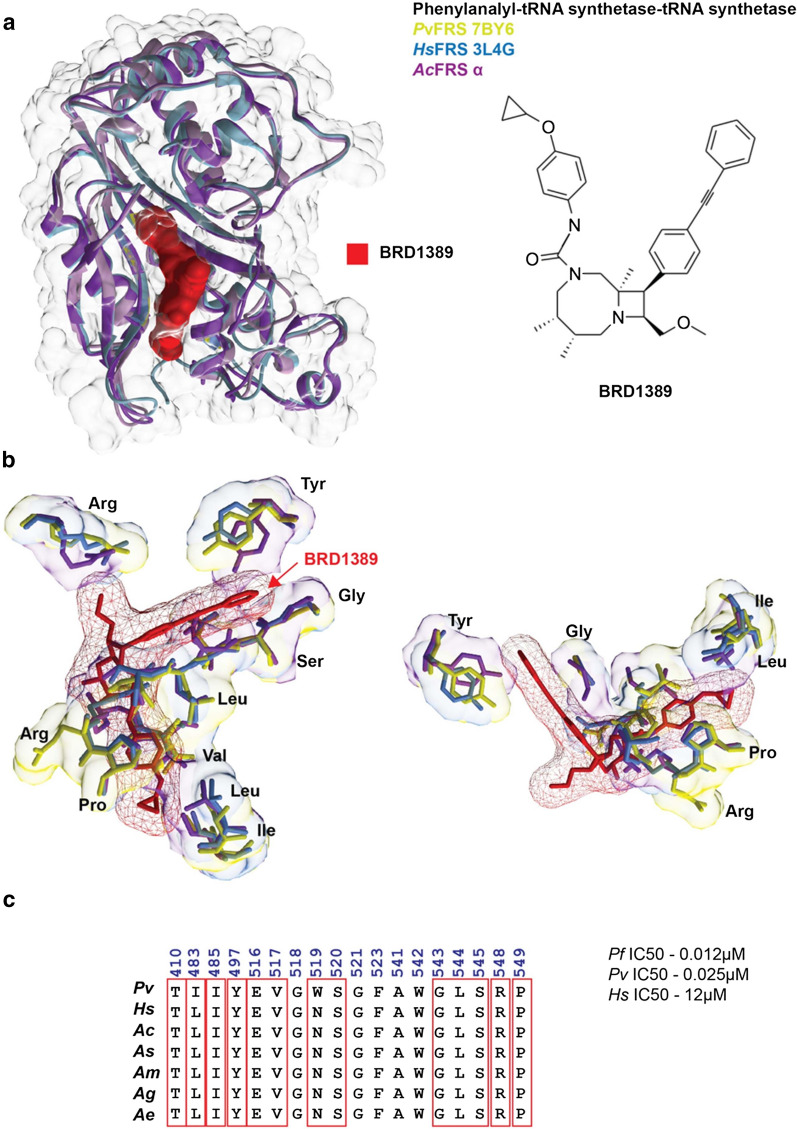
The structural analysis of the binding site in the phenylanalyl-tRNA synthetase from *An. culicifacies* in comparison with *P. vivax* and *H. sapiens.* **a** The three-dimensional crystal structure of the superimposed phenylanalyl-tRNA synthetase from *An. culicifacies* (built structure model, this study), *P. vivax* (PDB ID: 7BYG), and *H. sapiens* (PDB ID: 3L4G) are shown with bound inhibitor BRD1389 (in red). In the same figure chemical structure of the inhibitor BRD1389 is also depicted. **b** The key residues within 4 Å range of the BRD1389 binding cavity are shown for *Pv* (yellow), *Hs* (blue), and *Ac* (purple). **c** Multiple sequence alignment of the BRD1389 interacting residues is shown, with the key residues marked in red box. The IC_50_ value for the *Pf-*, *Pv-* and *Hs-*FRS are shown in µM range [35]. *Pv*: *P. vivax*; *Hs*: *H. sapiens*; *Ac*: *An. culicifacies*; *As*; *An. stephensi*; *Am*: *An. minimus*; *Ag*: *An. gambiae*; *Ae*: *Ae. aegypti*

Owing to increased insecticide resistance in several of the mosquito species, there is an immediate need to develop novel strategies to control mosquito populations. The availability of the genomes from several mosquito species has opened new avenues for screening novel insecticides. In the current study, we catalogued aaRSs from four different *Anopheles* spp. (*An. culicifacies*, *An. stephensi*, *An. gambiae*, and *An. minimus*) and *Ae. aegypti*. We show variation in the number of aaRSs present in the four *Anopheline* species compared to *Ae. aegypti*, as has been seen for several other species including *Plasmodium*, *H. sapiens*, and *Babesia* [36, 37]. In general, all organisms should have 20 aaRSs for protein synthesis (translation) coding for each of the amino acids, and an additional aaRSs enzyme is attributed to organelle-specific activity [53]. However, there are exceptions to this observation; for example, in bacteria and archaea families there are indirect routes of Gln-tRNA^Gln^ and Asn-tRNA^Asn^ synthesis that coexist in parallel with the classical synthesis pathways [54]. In addition, it was observed that organelle-specific tRNA synthetases remain missing (either partially or completely). Ideally, 20 different aaRSs are present within an organism/organelle, as stated earlier. However, occasionally the tRNAs and aaRSs are shared among more than one organelle, for example, FRS [36].

Among all aaRSs analyzed here, FRS is found to be the most interesting, as this is the only protein found in our analysis that consists of two chains, one α and one β, and similar to other species it likely exists as a heterodimer in solution [35]. It is worth noting that there are exceptions, and mitochondrial FRS (yeast and human) can exist as a monomer [55]. The α and β FRS subunits were found in the cytoplasm of all five studied mosquito species. Furthermore, a second copy of α-FRS was also detected in the mitochondria of all examined mosquito genomes. Our analysis of the three aaRSs—KRS, PRS, and FRS—also revealed partial structural conservation of the respective inhibitor binding site topology within the enzyme across species (Figs. 3b, 4b and Additional file 1: Fig. S1). We propose that antiplasmodial inhibitors like HF (PRS) and BRD1389 (FRS) may have the potential to be repurposed against *Anopheles* spp. and *Ae. aegypti*.

In conclusion, our analysis provides in-depth data on genome-wide identification and annotation of their potential localization along with domain arrangements of aaRSs from four different *Anopheles* spp. and *Ae. aegypti*. The aaRSs are essential for protein synthesis, and their inhibition is detrimental to the organism. AaRSs are a gene family that is considered a high-value drug target against parasites (especially protozoan parasites). Moderate sequence identity (40–80%) within the core synthetase domain of aaRSs from *H. sapiens* and mosquito species in our analysis suggests that targeting aaRSs in mosquito species can be effectively translated in designing safe (nontoxic) inhibitors against mosquitoes. Furthermore, the partial structural and sequence similarity between *Plasmodium* and mosquito aaRS binding sites offers a window for drug repurposing. Indeed, targeting any particular aaRS that is conserved between the mosquito (*Anopheles*) and the parasite (*Plasmodium*) presents a very unique opportunity. This comprehensive study of aaRSs from *Anopheles* and *Aedes* mosquitoes will be beneficial for new insecticide development and thus vector control via targeting aaRSs as potent insecticidal targets.

## Supplementary Information

**Additional file 1: Table S1.** Annotation of aminoacyl-tRNA synthetases (aaRSs) in five different mosquito genomes. Domain annotations were made using Pfam and InterPro. Physicochemical parameters of domains, for instance isoelectric points (pI), were calculated using ProtoParam. **Table S2.** Class I and II type aaRS average size (core synthetase domain) and isoelectric point. **Table S3.** Sequence-level comparison of core synthetase domain between human and *An. gambiae* and *An. culicifacies* aaRSs. **Figure S1.** Phylogenetic trees were constructed for three representative aminoacyl tRNA synthetases (lysyl-, KRS; prolyl-, PRS and phenylalanyl-, FRS). **a** For lysyl and prolyl aminoacyl tRNA synthetases, a phylogenetic tree was generated using the maximum likelihood method (MLM). **b** In the case of FRS, both alpha and beta subunits were considered for phylogenetic analysis. *Sc*: *Saccharomyces cerevisiae*; *Pf*: *P. falciparum*; *Hs*: *H. sapiens*; *Ec*: *Escherichia coli*; *Bv*: *Babesia bovis*; *Ae*: *Ae. aegypti*; *Ag*: *An. gambiae*; *Ac*: *An. culicifacies*; *As*: *An. stephensi*; *Am*: *An. minimus*; *Mtb*: *Mycobacterium tuberculosis*; *Pfu*: *Pyrococcus furiosus*.

## Data Availability

Individual aminoacyl-tRNA synthetase (aaRS) sequences, molecular weight, detailed domain diagrams including different domain parameters, and other materials are available from the corresponding author upon reasonable request.

## Abbreviations

aaRS: Aminoacyl-tRNA synthetase
DALR: [Aspartate (D), alanine (A), leucine (L), arginine (R)] anticodon binding domain (ABD) of arginyl tRNA synthetase
GST: Glutathione S-transferase
WHEP-TRS: Helix-turn-helix domain
ProRS-C_1: Prolyl-tRNA synthetase, C-terminal
FDX_ACB: Ferredoxin-fold anticodon binding domain
TGS: Threonyl-tRNA synthetase, GTPase, and SpoT
SAD: Second additional domain
DHHA: DHH associated domain
PheRS_DBD1: Phenylalanyl-tRNA synthetase DNA binding domain
HTS: Homoserine O-transsuccinylase domain
PhetRS_B1: Phe-tRNA synthetase beta subunit B1 domain
