# Supplementary material for: Drug targeting of aminoacyl-tRNA synthetases in Anopheles species and Aedes aegypti that cause malaria and dengue
Source: Parasit Vectors. 2021 Dec 11;14:605. doi: 10.1186/s13071-021-05106-5 (PMC8665550; doi:10.1186/s13071-021-05106-5)
Supplement: Supplementary file 1 — Additional file 1: Table S1. Annotation of aminoacyl-tRNA synthetases (aaRSs) in five different mosquito genomes. Domain annotations were made using Pfam and InterPro. Physicochemical parameters of domains, for instance isoelectric points (pI), were calculated using ProtoParam. Table S2. Class I and II type aaRS average size (core synthetase domain) and isoelectric point. Table S3. Sequence-level comparison of core synthetase domain between human and An. gambiae and An. culicifacies aaRSs. Figure S1. Phylogenetic trees were constructed for three representative aminoacyl tRNA synthetases (lysyl-, KRS; prolyl-, PRS and phenylalanyl-, FRS). a For lysyl and prolyl aminoacyl tRNA synthetases, a phylogenetic tree was generated using the maximum likelihood method (MLM). b In the case of FRS, both alpha and beta subunits were considered for phylogenetic analysis. Sc: Saccharomyces cerevisiae; Pf: P. falciparum; Hs: H. sapiens; Ec: Escherichia coli; Bv: Babesia bovis; Ae: Ae. aegypti; Ag: An. gambiae; Ac: An. culicifacies; As: An. stephensi; Am: An. minimus; Mtb: Mycobacterium tuberculosis; Pfu: Pyrococcus furiosus. [file 13071_2021_5106_MOESM1_ESM.docx]

**Electronic Supplementary Information**

**Parasites & Vector**

**Drug targeting aminoacyl-tRNA synthetases in *Anopheles* spp. and *Aedes aegypti* that cause malaria and dengue**

**Soumyananda Chakraborti ^a,1*^, Jyoti Chhibber‑Goel ^b,1^, and Amit Sharma^a,b^***

^a^National Institute of Malaria Research, New Delhi, India, ^b^ Molecular Medicine, International Centre for Genetic Engineering and Biotechnology, New Delhi, India

^1^ Authors have equal contribution

*Correspondence: soumyabiochem@gmail.com

| Type-I aaRSs domain in the genome of *Aedes aegyptie* | | | | | | |
| --- | --- | --- | --- | --- | --- | --- |
| **Class** | **Protein name** | **Vector Base ID** | **Localization** | **Length** | **pI** | **Domain Details** |
| I | LRS | AAEL006415 | Cytoplasm | 1182 | 6.9 | 1. t-RNA_Synt_1  2. anticodon binding domain |
| I | LRS | AAEL007130 | Mitochondria | 896 | 7.8 | 1. t-RNA_Synt_1  2. anticodon binding domain |
| I | IRS | AAEL000388 | Cytoplasmic | 1226 | 8.3 | 1. t-RNA_Synt_1  2. anticodon binding domain |
| I | IRS* | AAEL020387 | Mitochondria | 961 | 8.2 | 1. t-RNA_Synt_1  2. anticodon binding domain |
| I | VRS | AAEL004327 | Cytoplasmic | 1048 | 6.1 | 1. t-RNA_Synt_1  2. anticodon binding domain  3. Val_t-RNA synthatase arm |
| I | VRS | AAEL014099 | Cytoplasmic | 980 | 8.7 | 1. t-RNA_Synt_1  2. anticodon binding domain |
| I | RRS | AAEL000199  **Table S1**. Tentative annotation of Aminoacyl-tRNA synthetases (aaRSs) in five different mosquito genome. Domain annotations were made using pfam and InterPro. Physicochemical parameters of domains for instance iso-electric points (pI) were calculated using ProtoParam. | Cytoplasmic | 671 | 6.7 | 1. Arg_t-RNA_Synt_N  2. t-RNA_Synt_1  3. DALR_1 |
| I | RRS** | AAEL013862 | Mitochondria | 557 | 8.7 | 1. t-RNA_Synt_1  2. DALR_1 |
| I | CRS | AAEL004345 | Cytoplasmic | 735 | 5.9 | 1. t-RNA_Synt_1  2. anticodon binding domain |
| I | CRS | AAEL022886 | Mitochondria | 513 | 7.6 | 1. t-RNA_Synt_1  2. anticodon binding domain |
| I | WRS | AAEL013521 | Cytoplasmic | 417 | 6.1 | 1. t-RNA_Synt_1 |
| I | WRS | AAEL006769 | Mitochondria | 375 | 8.6 | 1. t-RNA_Synt_1 |
| I | MRS | AAEL014709 | Cytoplasmic | 966 | 8.2 | 1. GST_N_5  2. GST_C_3  3. t-RNA_Synt_1  4. WHEP-TRS (2 copies) |
| I | MRS | AAEL003913 | Mitochondria | 553 | 8.3 | 1. t-RNA_Synt_1  2. anticodon binding domain |
| I | YRS | AAEL001044 | Cytoplasmic | 524 | 6.4 | 1. t-RNA_Synt_1  2. anticodon binding domain |
| I | YRS | AAEL010563 | Mitochondria | 463 | 9.1 | 1. t-RNA_Synt_1 |
| I | ERS | AAEL005688 | Mitochondria | 527 | 9.1 | 1. t-RNA_Synt_1  2. anticodon binding domain |
| I | QRS | AAEL011395 | Cytoplasmic | 782 | 6.0 | 1. t-RNA_synthatase_1c_R1  2. t-RNA_synthatase_1c_R2  3. t-RNA_Synt_1  4. t-RNA_Synt_1c_C |

| Type-II aaRSs domain in the genome of *Aedes aegyptie* | | | | | | |
| --- | --- | --- | --- | --- | --- | --- |
| **Class** | **Protein name** | **Vector Base ID** | **Localization** | **Length** | **pI** | **Domain Details** |
| II | ARS | AAEL008509 | Mitochondria | 1012 | 7.0 | 1. t-RNA_synthatase_2  2. t-RNA SAD |
| II | ARS | AAEL001218 | Cytoplasm | 966 | 5.7 | 1. t-RNA_synthatase_2  2. t-RNA SAD  3. DHHA1 |
| II | GRS*** | AAEL005728 | Mitochondria | 758 | 7.8 | 1. t-RNA_Synt_2  2. anticodon binding domain  3. WHEP-TRS |
| II | FRS (Alpha) | AAEL002867 | Cytoplasmic | 496 | 6.3 | 1. PheRS_DBD1  2. PheRS_DBD2  3. PheRS_DBD3  4. t-RNA_Synt_2 |
| II | FRS (Alpha) | AAEL003994 | Mitochondria | 445 | 7.0 | 1. t-RNA_Synt_2  2. FDX_ACB |
| II | FRS (Beta) | AAEL004613 | Cytoplasmic | 590 | 5.8 | 1. PhetRS_B1  2. B3_B4  3.B5  4. t-RNA_synthFbeta |
| II | HRS**** | AAEL002048 | Cytoplasmic | 514 | 6.1 | 1. t-RNA_Synt_2  2. anticodon binding domain (HGTP)  3. WHEP-TRS |
| II | KRS**** | AAEL014702 | Cytoplasmic | 588 | 6.0 | 1. t-RNA_synthatase_2  2. anticodon binding domain |
| II | PRS | AAEL010816 | Mitochondria | 435 | 7.2 | 1. t-RNA_synthatase_2  2. anticodon binding domain (HGTP) |
| II | NRS^#^ | AAEL006577 | Cytoplasmic | 553^#^ | 5.7 | 1. t-RNA_synthatase_2  2. anticodon binding domain |
| II | NRS^#^ | AAEL011655 | Mitochondria | 480 | 7.0 | 1. t-RNA_synthatase_2  2. anticodon binding domain |
| II | DRS | AAEL001200 | Mitochondria | 682 | 8.0 | 1. t-RNA_synthatase_2  2. anticodon binding domain |
| II | DRS | AAEL002748 | Cytoplasmic | 534 | 6.7 | 1. t-RNA_synthatase_2  2. anticodon binding domain |
| II | SRS | AAEL005037 | Cytoplasmic | 500 | 6.0 | 1. Seryl_t-RNA_N  2. t-RNA_synthatase_2 |
| II | SRS | AAEL005831 | Mitochondria | 462 | 8.2 | 1. t-RNA_synthatase_2 |
| II | TRS | AAEL001206 | Cytoplasmic | 754 | 6.9 | 1. TGS  2. t-RNA SAD  3. t-RNA_synthatase_2  4. anticodon binding domain (HGTP) |

| Bifunctional aaRSs domain in the genome of *Aedes aegyptie* | | | | | | |
| --- | --- | --- | --- | --- | --- | --- |
| **Class** | **Protein name** | **Vector Base ID** | **Localization** | **Length** | **pI** | **Domain Details** |
| I,II | EPRS  PRS, ERS | AAEL023850 | Cytoplasmic | 1638 | 8.6 | 1. t-RNA_synthatase_1c_C  2. t-RNA_synthatase_1c_anti-codon  3. WHEP-TRS (total 5 domain)  4. t-RNA_synthatase_2  5. anticodon binding domain (HGTP)  6. prolyl t-RNA synthatase C-terminal |

* According to VectorBase annotation Unspecific product however 60% identity with Ag-IRS, ID: AP003156.

**According to DeepLock 1.0 RRS is localized in Cytoplasm

*** Unspecific product according to VB however 90% identity with Ag-GRS, ID: AGAP008604, except N-terminus.

**** Only one copy (KRS and HRS) in rest of the mosquitoes 2 copies.

^#^ annotated as Aspartyl in VB

| Type-I aaRSs domain in the genome of *Anopheles gambiae* | | | | | | |
| --- | --- | --- | --- | --- | --- | --- |
| **Class** | **Protein name** | **Vector Base ID** | **Localization** | **Length** | **pI** | **Domain Details** |
| I | LRS | AGAP008297 | Cytoplasm | 1195 | 7.9 | 1. t-RNA_Synt_1  2. anticodon binding domain |
| I | LRS | AGAP002014 | Mitochondria | 887 | 7.8 | 1. t-RNA_Synt_1  2. anticodon binding domain |
| I | IRS | AGAP002101 | Cytoplasmic | 1217 | 7.5 | 1. t-RNA_Synt_1  2. anticodon binding domain |
| I | IRS | AGAP003156 | Mitochondria | 970 | 8.3 | 1. t-RNA_Synt_1  2. anticodon binding domain |
| I | VRS | AGAP010420 | Cytoplasmic | 1048 | 7.9 | 1. t-RNA_Synt_1  2. anticodon binding domain  3.Val_t-RNA synthatase arm |
| I | VRS | AGAP006624 | Mitochondria | 993 | 7.8 | 1. t-RNA_Synt_1  2. anticodon binding domain |
| I | RRS | AGAP004708 | Cytoplasmic | 663 | 6.3 | 1. Arg_t-RNA_Synt_N  2. t-RNA_Synt_1  3. DALR_1 |
| I | RRS* | AGAP010086 | Mitochondria | 569 | 7.9 | 1. t-RNA_Synt_1  2. DALR_1 |
| I | CRS | AGAP012283 | Cytoplasmic | 743 | 5.9 | 1. t-RNA_Synt_1  2. anticodon binding domain |
| I | CRS | AGAP011821 | Mitochondria | 523 | 8.0 | 1. t-RNA_Synt_1  2. anticodon binding domain |
| I | WRS | AGAP003315 | Cytoplasmic | 444 | 6.7 | 1. t-RNA_Synt_1 |
| I | WRS | AGAP011744 | Mitochondria | 375 | 8.9 | 1. t-RNA_Synt_1 |
| I | MRS | AGAP007891 | Cytoplasmic | 999 | 8.5 | 1. GST_N_5  2. GST_C_3  3. t-RNA_Synt_1  4. WHEP-TRS (2 copies) |
| I | MRS | AGAP002383 | Mitochondria | 563 | 7.3 | 1. t-RNA_Synt_1  2. anticodon binding domain |
| I | YRS | AGAP003003 | Cytoplasmic | 542 | 6.4 | 1. t-RNA_Synt_1  2. t-RNA-binding domain |
| I | YRS** | AGAP000012 | Mitochondria | 463 | 9.3 | 1. t-RNA_Synt_1 |
| I | ERS | AGAP002570 | Mitochondrial | 546 | 9.2 | 1. t-RNA_Synt_1  2. anticodon binding domain |
| I | QRS | AGAP010267 | Cytoplasmic | 772 | 6.7 | 1. t-RNA_synthatase_1c_R1  2. t-RNA_synthatase_1c_R2  3. t-RNA_Synt_1  4. t-RNA_Synt_1c_anti-codon |

| Type-II aaRSs domain in the genome of *Anopheles gambiae* | | | | | | |
| --- | --- | --- | --- | --- | --- | --- |
| **Class** | **Protein name** | **Vector Base ID** | **Localization** | **Length** | **pI** | **Domain Details** |
| II | ARS | AGAP006997 | Mitochondria | 1011 | 8.0 | 1. t-RNA_synthatase_2  2. t-RNA SAD |
| II | ARS | AGAP009701 | Cytoplasm | 967 | 5.8 | 1. t-RNA_synthatase_2  2. t-RNA SAD  3. DHHA1 |
| II | GRS | AGAP008604 | Cytoplasm | 679 | 6.1 | 1. t-RNA_Synt_2  2. anticodon binding domain  3. WHEP-TRS |
| II | FRS (Alpha) | AGAP002653 | Cytoplasmic | 496 | 6.9 | 1. PheRS_DBD1  2. PheRS_DBD2  3. PheRS_DBD3  4. t-RNA_Synt_2 |
| II | FRS (Alpha) | AGAP012096 | Mitochondria | 465 | 7.1 | 1. t-RNA_Synt_2  2. FDX_ACB |
| II | FRS (Beta) | AGAP003517 | Cytoplasmic | 590 | 6.2 | 1. PhetRS_B1  2. B3_B4  3.B5  4. t-RNA_synthFbeta |
| II | HRS*** | AGAP000735 | Cytoplasmic | 533 | 6.1 | 1. t-RNA_Synt_2  2. anticodon binding domain (HGTP)  3. WHEP-TRS |
| II | HRS*** | AGAP002294 | Mitochondria | 513 | 6.9 | 1. t-RNA_Synt_2  2. anticodon binding domain (HGTP) |
| II | KRS | AGAP000325 | Cytoplasmic | 586 | 5.7 | 1. t-RNA_synthatase_2  2. anticodon binding domain |
| II | KRS | AGAP007858 | Cytoplasmic | 575 | 6.1 | 1. t-RNA_synthatase_2  2. anticodon binding domain |
| II | PRS | AGAP003589 | Mitochondria | 461 | 8.2 | 1. t-RNA_synthatase_2  2. anticodon binding domain (HGTP) |
| II | NRS | AGAP002969 | Cytoplasmic | 553^#^ | 5.6 | 1. t-RNA_synthatase_2  2. anticodon binding domain |
| II | NRS | AGAP001276 | Mitochondria | 474 | 7.6 | 1. t-RNA_synthatase_2  2. anticodon binding domain |
| II | DRS | AGAP007844 | Mitochondria | 683 | 8.6 | 1. t-RNA_synthatase_2  2. anticodon binding domain |
| II | DRS | AGAP005576 | Cytoplasmic | 543 | 6.5 | 1. t-RNA_synthatase_2  2. anticodon binding domain |
| II | SRS | AGAP008265 | Cytoplasmic | 503 | 6.0 | 1. Seryl_t-RNA_N  2. t-RNA_synthatase_2 |
| II | SRS**** | AGAP000991 | Mitochondria | 433 | 8.5 | 1. t-RNA_synthatase_2 |
| II | TRS***** | AGAP008929 | Cytoplasmic | 767 | 7.9 | 1. TGS  2. t-RNA SAD  3. t-RNA_synthatase_2  4. anticodon binding domain (HGTP) |

| Bi-functional aaRSs domain in the genome of *Anopheles gambiae* | | | | | | |
| --- | --- | --- | --- | --- | --- | --- |
| **Class** | **Protein name** | **Vector Base ID** | **Localization** | **Length** | **pI** | **Domain Details** |
| I,II | EPRS  PRS, ERS | AGAP002945 | Cytoplasmic | 1813 | 8.7 | 1. t-RNA_synthatase_1c  2. t-RNA_synthatase_1c_anti-codon  3. WHEP-TRS (total 7 domain)  4. t-RNA_synthatase_2  5. anticodon binding domain (HGTP)  6. prolyl t-RNA synthatase C-terminal |

*According to Deeplock 1.0 RRS is localized in Cytoplasm

**According to Uniprot YRS is localized in both Cytoplasm and Mitochondria

***Comparison of cytoplasm and mitochondrial variant of HRS shows differences in N and C terminus only, rest of the protein are similar.

**** According to Deeplock 1.0 SRS is localized in both Cytoplasm and Mitochondria

*****According to Deeplock 1.0TRS is localized in Mitochondria

| Type-I aaRSs domain in the genome of *Anopheles minimus* | | | | | | |
| --- | --- | --- | --- | --- | --- | --- |
| **Class** | **Protein name** | **Vector Base ID** | **Localization** | **Length** | **pI** | **Domain Details** |
| I | LRS* | AMIN006003 | Cytoplasm | 1673 | 6.7 | 1. t-RNA_Synt_1  2. anticodon binding domain |
| I | LRS | AMIN003325 | Mitochondria | 909 | 7.3 | 1. t-RNA_Synt_1  2. anticodon binding domain |
| I | IRS | AMIN000637 | Cytoplasmic | 1217 | 7.5 | 1. t-RNA_Synt_1  2. anticodon binding domain |
| I | IRS | AMIN004624 | Mitochondria | 969 | 8.6 | 1. t-RNA_Synt_1  2. anticodon binding domain |
| I | VRS | AMIN004974 | Cytoplasmic | 1046 | 7.3 | 1. t-RNA_Synt_1  2. anticodon binding domain  3.Val_t-RNA synthatase arm |
| I | VRS | AMIN007771 | Mitochondria | 992 | 8.5 | 1. t-RNA_Synt_1  2. anticodon binding domain |
| I | RRS** | AMIN007476 | Cytoplasmic | 700 | 7.2 | 1. Arg_t-RNA_Synt_N  2. t-RNA_Synt_1  3. DALR_1 |
| I | RRS** | AMIN006642 | Mitochondria | 593 | 7.07 | 1. t-RNA_Synt_1  2. DALR_1 |
| I | CRS | AMIN006892 | Cytoplasmic | 744 | 5.8 | 1. t-RNA_Synt_1  2. anticodon binding domain |
| I | CRS*** | AMIN004326 | Mitochondria | 1338 | 6.8 | 1. t-RNA_Synt_1  2. anticodon binding domain |
| I | WRS**** | AMIN002839 | Cytoplasmic | 788 | 6.1 | 1. t-RNA_Synt_1 |
| I | WRS**** | AMIN008713 | Mitochondria | 1324 | 9.0 | 1. t-RNA_Synt_1 |
| I | MRS | AMIN007112 | Cytoplasmic | 996 | 8.5 | 1. GST_N_5  2. GST_C_3  2. t-RNA_Synt_1  3. WHEP-TRS (2 copies) |
| I | MRS | AMIN004672 | Mitochondria | 560 | 8.9 | 1. t-RNA_Synt_1  2. t-RNA-binding domain |
| I | YRS | AMIN003711 | Cytoplasmic | 542 | 6.2 | 1. t-RNA_Synt_1  2. t-RNA-binding domain |
| I | YRS | AMIN002078 | Mitochondria | 463 | 9.1 | 1. t-RNA_Synt_1 |
| I | ERS | AMIN002578 | Mitochondrial | 543 | 9.0 | 1. t-RNA_Synt_1  2. anticodon binding domain |
| I | QRS | AMIN001124 | Cytoplasmic | 771 | 6.8 | 1. t-RNA_synthatase_1c_R1  2. t-RNA_synthatase_1c_R2  3. t-RNA_Synt_1  4. t-RNA_Synt_1c_anti-codon |

| Type-II aaRSs domain in the genome of *Anopheles minimus* | | | | | | |
| --- | --- | --- | --- | --- | --- | --- |
| **Class** | **Protein name** | **Vector Base ID** | **Localization** | **Length** | **pI** | **Domain Details** |
| II | ARS***** | AMIN009982 | Mitochondria | 1009 | 7.0 | 1. t-RNA_synthatase_2  2. t-RNA SAD |
| II | ARS | AMIN009451 | Cytoplasm | 967 | 5.9 | 1. t-RNA_synthatase_2  2. t-RNA SAD  3. DHHA1 |
| II | GRS | AMIN009142 | Cytoplasm | 679 | 5.9 | 1. t-RNA_Synt_2  2. anticodon binding domain  3. WHEP-TRS |
| II | FRS (Alpha) | AMIN003649 | Cytoplasmic | 496 | 6.4 | 1. PheRS_DBD1  2. PheRS_DBD2  3. PheRS_DBD3  4. t-RNA_Synt_2 |
| II | FRS (Alpha) | AMIN008263 | Mitochondria | 461 | 7.1 | 1. t-RNA_Synt_2  2. FDX_ACB |
| II | FRS (Beta) | AMIN000880 | Cytoplasmic | 590 | 6.2 | 1. PhetRS_B1  2. B3_B4  3.B5  4. t-RNA_synthFbeta |
| II | HRS | AMIN005167 | Cytoplasmic | 521 | 5.8 | 1. t-RNA_Synt_2  2. anticodon binding domain (HGTP)  3. WHEP-TRS |
| II | HRS | AMIN004573 | Cytoplasmic | 473 | 7.6 | 1. t-RNA_Synt_2  2. anticodon binding domain (HGTP) |
| II | KRS****** | AMIN005476 | Cytoplasmic | 862 | 7.2 | 1. t-RNA_synthatase_2  2. anticodon binding domain |
| II | KRS | AMIN004336 | Cytoplasmic | 592 | 5.9 | 1. t-RNA_synthatase_2  2. anticodon binding domain |
| II | PRS | AMIN015785 | Mitochondria | 459 | 8.0 | 1. t-RNA_synthatase_2  2. anticodon binding domain (HGTP) |
| II | NRS | AMIN015873 | Cytoplasmic | 553^#^ | 5.6 | 1. t-RNA_synthatase_2  2. anticodon binding domain |
| II | NRS | AMIN002206 | Mitochondria | 471 | 7.0 | 1. t-RNA_synthatase_2  2. anticodon binding domain |
| II | DRS | AMIN007164 | Mitochondria | 677 | 8.4 | 1. t-RNA_synthatase_2  2. anticodon binding domain |
| II | DRS | AMIN010888 | Cytoplasmic | 543 | 6.6 | 1. t-RNA_synthatase_2  2. anticodon binding domain |
| II | SRS | AMIN015918 | Cytoplasmic | 503 | 6.00 | 1. Seryl_t-RNA_N  2. t-RNA_synthatase_2 |
| II | SRS | AMIN001949 | Mitochondria | 429 | 8.4 | 1. t-RNA_synthatase_2 |
| II | TRS******* | AMIN005887 | Cytoplasmic | 1021 | 8.5 | 1. TGS  2. t-RNA SAD  3. t-RNA_synthatase_2  4. anticodon binding domain (HGTP) |

| Bi-functional aaRSs domain in the genome of *Anopheles minimus* | | | | | | |
| --- | --- | --- | --- | --- | --- | --- |
| **Class** | **Protein name** | **Vector Base ID** | **Localization** | **Length** | **pI** | **Domain Details** |
| I,II | ( EPRS) ********  PRS, ERS | AMIN003171 | Cytoplasmic | 2335 | 7.8 | 1. t-RNA_synthatase_1c  2. t-RNA_synthatase_1c_anti-codon  3. WHEP-TRS (total 6 domain)  4. t-RNA_synthatase_2  5. anticodon binding domain (HGTP)  6. prolyl t-RNA synthatase C-terminal |

*LRS, N-terminal is long compared to other anopheles and contains completely unrelated Trypsin domin

**According to DeepLock 1.0 RRS (AMIN007476) is localized in mitochondria and RRS (AMIN006642) is localized in cytoplasm

*** CRS is having additional 815 residues at N-terminus, according to DeepLock 1.0 is localized in cytoplasm

**** Both the variants of WRS are having very long and unrelated C-terminal, rest of the protein is similar to other anopheles.

***** According to Uniprot ARS is localized in cytoplsm

******KRS C-terminal is unrelated to others

******* In TRS first 770 residues are almost same with other anopheles however C-terminal is long and not comparable to others and contains PIG-X domain

******** In EPRS N-(first 633 residues) and C-terminals are completely different from other anopheles spp. and also contains SMC_N domain at the N-Terminus

| Type-I aaRSs domain in the genome of *Anopheles Culicifacies* | | | | | | |
| --- | --- | --- | --- | --- | --- | --- |
| **Class** | **Protein name** | **Vector Base ID** | **Localization** | **Length** | **pI** | **Domain Details** |
| I | LRS | ACUA010057 | Cytoplasm | 1194 | 7.7 | 1. t-RNA_Synt_1  2. anticodon binding domain |
| I | LRS | ACUA007348 | Mitochondria | 909 | 7.1 | 1. t-RNA_Synt_1  2. anticodon binding domain |
| I | IRS | ACUA012116 | Cytoplasmic | 1217 | 8.1 | 1. t-RNA_Synt_1  2. anticodon binding domain |
| I | IRS | ACUA003704 | Mitochondria | 969 | 8.2 | 1. t-RNA_Synt_1  2. anticodon binding domain |
| I | VRS | ACUA008589 | Cytoplasmic | 1122 | 7.0 | 1. t-RNA_Synt_1  2. anticodon binding domain |
| I | VRS | ACUA012391 | Cytoplasmic | 1046 | 7.71 | 1. t-RNA_Synt_1  2. anticodon binding domain  3. Val_t-RNA synthatase arm |
| I | RRS | ACUA016164 | Cytoplasmic | 663 | 6.3 | 1. Arg_t-RNA_Synt_N  2. t-RNA_Synt_1  3. DALR_1 |
| I | RRS* | ACUA001323 | Mitochondria | 569 | 8.1 | 1. t-RNA_Synt_1  2. DALR_1 |
| I | CRS | ACUA005881 | Cytoplasmic | 744 | 5.7 | 1. t-RNA_Synt_1  2. anticodon binding domain |
| I | CRS | ACUA025758 | Mitochondria | 524 | 7.4 | 1. t-RNA_Synt_1  2. anticodon binding domain |
| I | WRS | ACUA015360 | Cytoplasmic | 445 | 6.5 | 1. t-RNA_Synt_1 |
| I | WRS | ACUA017755 | Mitochondria | 355 | 7.1 | 1. t-RNA_Synt_1 |
| I | MRS | ACUA016678 | Cytoplasmic | 998 | 8.3 | 1. GST_N_5  2. GST_C_3  3. t-RNA_Synt_1  4. WHEP-TRS (2 copies) |
| I | MRS | ACUA005528 | Mitochondria | 537 | 7.7 | 1. t-RNA_Synt_1  2. anticodon binding domain |
| I | YRS | ACUA023263 | Cytoplasmic | 542 | 6.2 | 1. t-RNA_Synt_1  2. anticodon binding domain |
| I | YRS** | ACUA017779 | Mitochondria | 728 | 6.9 | 1. t-RNA_Synt_1 |
| I | ERS | ACUA027793 | Mitochondrial | 543 | 9.1 | 1. t-RNA_Synt_1  2. anticodon binding domain |
| I | QRS | ACUA019275 | Cytoplasmic | 773 | 6.5 | 1. t-RNA_synthatase_1c_R1  2. t-RNA_synthatase_1c_R2  3. t-RNA_Synt_1  4. t-RNA_Synt_1c_anti-codon |

| Type-II aaRSs domain in the genome of *Anopheles Culicifacies* | | | | | | |
| --- | --- | --- | --- | --- | --- | --- |
| **Class** | **Protein name** | **Vector Base ID** | **Localization** | **Length** | **pI** | **Domain Details** |
| II | ARS | ACUA024643 | Mitochondria | 1012 | 6.7 | 1. t-RNA_synthatase_2  2. t-RNA SAD |
| II | ARS | ACUA022424 | Cytoplasm | 967 | 6.0 | 1. t-RNA_synthatase_2  2. t-RNA SAD  3. DHHA1 |
| II | GRS*** | ACUA019590 | Cytoplasm | 782 | 8.2 | 1. t-RNA_Synt_2  2. anticodon binding domain  3. WHEP-TRS |
| II | FRS (Alpha) | ACUA017843 | Cytoplasmic | 496 | 6.7 | 1. PheRS_DBD1  2. PheRS_DBD2  3. PheRS_DBD3  4. t-RNA_Synt_2 |
| II | FRS (Alpha) | ACUA012073 | Mitochondria | 461 | 7.3 | 1. t-RNA_Synt_2  2. FDX_ACB |
| II | FRS (Beta) | ACUA007664 | Cytoplasmic | 590 | 5.9 | 1. PhetRS_B1  2. B3_B4  3.B5  4. t-RNA_synthFbeta |
| II | HRS | ACUA013875 | Mitochondrial | 546 | 6.3 | 1. t-RNA_Synt_2  2. anticodon binding domain (HGTP) |
| II | HRS | ACUA018854 | Cytoplasmic | 475 | 6.3 | 1. t-RNA_Synt_2  2. anticodon binding domain (HGTP) |
| II | KRS**** | ACUA027039^#^ | Cytoplasmic | 592 | 5.8 | 1. t-RNA_synthatase_2  2. anticodon binding domain |
| II | KRS**** | ACUA022438^#^ | Cytoplasmic | 541 | 7.3 | 1. t-RNA_synthatase_2  2. anticodon binding domain |
| II | PRS | ACUA009586 | Mitochondria | 459 | 7.6 | 1. t-RNA_synthatase_2  2. anticodon binding domain (HGTP) |
| II | NRS | ACUA008747 | Cytoplasmic | 553 | 5.7 | 1. t-RNA_synthatase_2  2. anticodon binding domain |
| II | NRS | ACUA021747 | Mitochondria | 471 | 6.6 | 1. t-RNA_synthatase_2  2. anticodon binding domain |
| II | DRS***** | ACUA021543 | Mitochondria | 585 | 7.0 | 1. t-RNA_synthatase_2  2. anticodon binding domain |
| II | DRS | ACUA019396 | Cytoplasmic | 538 | 6.3 | 1. t-RNA_synthatase_2  2. anticodon binding domain |
| II | SRS | ACUA017653 | Cytoplasmic | 503 | 6.0 | 1. Seryl_t-RNA_N  2. t-RNA_synthatase_2 |
| II | SRS | ACUA016890 | Mitochondria | 440 | 7.9 | 1. t-RNA_synthatase_2 |
| II | TRS ****** | ACUA011960 | Cytoplasmic | 768 | 8.1 | 1. TGS  2. t-RNA SAD  3. t-RNA_synthatase_2  4. anticodon binding domain (HGTP) |

| Bi-functional aaRSs domain in the genome of *Anopheles Culicifacies* | | | | | | |
| --- | --- | --- | --- | --- | --- | --- |
| **Class** | **Protein name** | **Vector Base ID** | **Localization** | **Length** | **pI** | **Domain Details** |
| I,II | ( EPRS)  PRS, ERS | ACUA007117 | Cytoplasmic | 1722 | 8.8 | 1. t-RNA_synthatase_1c  2. t-RNA_synthatase_1c_anti-codon  3. WHEP-TRS (total 6 domain)  4. t-RNA_synthatase_2  5. anticodon binding domain (HGTP)  6. prolyl t-RNA synthatase C-terminal |

*According to DeepLock 1.0 RRS is localized in cytoplasm

** YRS is bigger compared to all other anopheles as its C-terminus is extra-long and contains unrelated popeye domain.

*** According to DeepLock 1.0 GRS is localized in mitochondria

**** The only difference in KRS I and II is the c-terminus; first 481 residues are almost identical.

*****According to Uniprot DRS is localized in cytoplasm

******According to DeepLock 1.0 TRS is localized in mitochondria

| Type-I aaRSs domain in the genome of *Anopheles stephensi* | | | | | | |
| --- | --- | --- | --- | --- | --- | --- |
| **Class** | **Protein name** | **Vector Base ID** | **Localization** | **Length** | **pI** | **Domain Details** |
| I | LRS | ASTEI02989 | Cytoplasm | 1188 | 7.9 | 1. t-RNA_Synt_1  2. anticodon binding domain |
| I | LRS | ASTEI00364 | Mitochondria | 854 | 6.7 | 1. t-RNA_Synt_1  2. anticodon binding domain |
| I | IRS | ASTEI00404 | Cytoplasmic | 1219 | 7.7 | 1. t-RNA_Synt_1  2. anticodon binding domain |
| I | IRS | ASTEI00219 | Mitochondria | 969 | 8.4 | 1. t-RNA_Synt_1  2. anticodon binding domain |
| I | VRS | ASTEI09468 | Cytoplasmic | 1045 | 7.7 | 1. t-RNA_Synt_1  2. anticodon binding domain  3. Val_t-RNA synthatase arm |
| I | VRS | ASTEI02669 | Mitochondria | 997 | 8.5 | 1. t-RNA_Synt_1  2. anticodon binding domain |
| I | RRS | ASTEI08159 | Cytoplasmic | 762 | 6.5 | 1. Arg_t-RNA_Synt_N  2. t-RNA_Synt_1  3. DALR_1 |
| I | RRS | ASTE008389^#^ | Mitochondria | 561 | 7.9 | 1. t-RNA_Synt_1  2. DALR_1 |
| I | CRS | ASTEI02422 | Cytoplasmic | 745 | 5.9 | 1. t-RNA_Synt_1  2. anticodon binding domain |
| I | CRS* | ASTEI09229 | Mitochondria | 1347 | 6.5 | 1. t-RNA_Synt_1  2. anticodon binding domain |
| I | WRS | ASTEI00914 | Cytoplasmic | 445 | 6.5 | 1. t-RNA_Synt_1 |
| I | WRS | ASTEI09168 | Mitochondria | 346 | 7.1 | 1. t-RNA_Synt_1 |
| I | MRS | ASTEI07715 | Cytoplasmic | 995 | 8.5 | 1. GST_N_5  2. GST_C_3  3. t-RNA_Synt_1  4. WHEP-TRS (2 copies) |
| I | MRS | ASTEI04310 | Mitochondria | 563 | 8.0 | 1. t-RNA_Synt_1  2. 2. anticodon binding domain |
| I | YRS | ASTEI03284 | Cytoplasmic | 545 | 6.4 | 1. t-RNA_Synt_1  2. t-RNA-binding domain |
| I | YRS | ASTE007470^#^ | Mitochondria | 464 | 9.2 | 1. t-RNA_Synt_1 |
| I | ERS | ASTEI04043 | Mitochondrial | 544 | 9.1 | 1. t-RNA_Synt_1  2. anticodon binding domain |
| I | QRS | ASTEI11163 | Cytoplasmic | 771 | 6.5 | 1. t-RNA_synthatase_1c_R1  2. t-RNA_synthatase_1c_R2  3. t-RNA_Synt_1  4. t-RNA_Synt_1c_anti-codon |

| Type-II aaRSs domain in the genome of *Anopheles stephensi* | | | | | | |
| --- | --- | --- | --- | --- | --- | --- |
| **Class** | **Protein name** | **Vector Base ID** | **Localization** | **Length** | **pI** | **Domain Details** |
| II | ARS | ASTEI02850 | Mitochondria | 1009 | 7.8 | 1. t-RNA_synthatase_2  2. t-RNA SAD |
| II | ARS | ASTEI01989 | Cytoplasm | 967 | 5.8 | 1. t-RNA_synthatase_2  2. t-RNA SAD  3. DHHA1 |
| II | GRS** | ASTEI10716 | Cytoplasm | 746 | 7.6 | 1. t-RNA_Synt_2  2. anticodon binding domain  3. WHEP-TRS |
| II | FRS (Alpha) | ASTEI04135 | Cytoplasmic | 492 | 6.2 | 1. PheRS_DBD1  2. PheRS_DBD2  3. PheRS_DBD3  4. t-RNA_Synt_2 |
| II | FRS (Alpha) | ASTEI06084 | Mitochondria | 463 | 7.1 | 1. t-RNA_Synt_2  2. FDX_ACB |
| II | FRS (Beta) | ASTEI09345 | Cytoplasmic | 603 | 6.6 | 1. PhetRS_B1  2. B3_B4  3.B5  4. t-RNA_synthFbeta |
| II | HRS | ASTEI07011 | Cytoplasmic | 500 | 5.6 | 1. t-RNA_Synt_2  2. anticodon binding domain (HGTP)  3. WHEP-TRS |
| II | HRS | ASTEI00171 | Cytoplasmic | 466 | 7.5 | 1. t-RNA_Synt_2  2. anticodon binding domain (HGTP) |
| II | KRS*** | ASTE001139^#^ | Mitochondria | 623 | 6.7 | 1. t-RNA_synthatase_2  2. anticodon binding domain |
| II | KRS*** | ASTEI08818^#^ | Cytoplasmic | 588 | 6.0 | 1. t-RNA_synthatase_2  2. anticodon binding domain |
| II | PRS**** | ASTEI04362 | Mitochondria | 527 | 8.6 | 1. t-RNA_synthatase_2  2. anticodon binding domain (HGTP) |
| II | NRS | ASTEI00518 | Cytoplasmic | 553 | 5.6 | 1. t-RNA_synthatase_2  2. anticodon binding domain |
| II | NRS | ASTEI05804 | Mitochondria | 472 | 6.9 | 1. t-RNA_synthatase_2  2. anticodon binding domain |
| II | DRS | ASTE002677 | Mitochondria | 637 | 8.0 | 1. t-RNA_synthatase_2 |
| II | DRS | ASTEI02180 | Cytoplasmic | 543 | 6.4 | 1. t-RNA_synthatase_2  2. anticodon binding domain |
| II | SRS | ASTEI03010 | Cytoplasmic | 503 | 5.9 | 1. Seryl_t-RNA_N  2. t-RNA_synthatase_2 |
| II | SRS | ASTEI03468 | Mitochondria | 444 | 8.2 | 1. t-RNA_synthatase_2 |
| II | TRS***** | ASTEI01924 | Cytoplasmic | 970 | 8.4 | 1. TGS  2. t-RNA SAD  3. t-RNA_synthatase_2  4. anticodon binding domain (HGTP) |

| Bi-functional aaRSs domain in the genome of *Anopheles stephensi* | | | | | | |
| --- | --- | --- | --- | --- | --- | --- |
| **Class** | **Protein name** | **Vector Base ID** | **Localization** | **Length** | **pI** | **Domain Details** |
| I,II | ( EPRS)  PRS, ERS | ASTEI00498 | Cytoplasmic | 1716 | 8.5 | 1. t-RNA_synthatase_1c  2. t-RNA_synthatase_1c_anti-codon  3. WHEP-TRS (total 6 domain)  4. t-RNA_synthatase_2  5. anticodon binding domain (HGTP)  6. prolyl t-RNA synthatase C-terminal |

*CRS is possessing an extra 821 residues at N-terminus. According to Deeplock 1.0 the protein is localized in cytoplasm and nucleus

**According to Deeplock 1.0 GRS is localized in Mitochondria

***Except first few residues in N-terminus, KRS-I and KRS-II are identical

**** According to Pfam PRS carries ATP_synt_H domain at N-terminus

*****In TRS C-terminal is long and unrelated and carries PIG-X domain compared to other mosquito species.

^#^SDA-500 (Pakistani strain, no good match found with Indian strain), according to Deeplock 1.0 RRS (ASTE008389) is localized both cytoplasm and mitochondria.

**Table S2:** Type-I and Type-II aaRSs average size (core synthatase domain) and iso-electric point.

| **Name** | **Type** | **Domain Length (range)** | **Iso-electric point (pI)** |
| --- | --- | --- | --- |
| Synthatase domain | Type-I | 281-740 | 5.2-9.2 |
|  | Type-II | 184-610 | 5.0-8.5* |

*In most cases pI is found in the range of 5 to 7.

**Table S3:** Tentative sequence comparison of core synthatase domain between human and *An. gambiae*, *An. culicifacies* aaRSs

| **Class** | **Protein name** | **Uniprot ID** | **Length** | **Similarity to *An. gambiae*** | **Similarity to *An. Culicifacies*** |
| --- | --- | --- | --- | --- | --- |
| I | LRS | Q9P2J5 | 1176 | 66.5 | 66.8 |
| I | LRS | Q15031 | 903 | 47.1 | 46.3 |
| I | IRS | P41252 | 1262 | 70.6 | 70.1 |
| I | IRS | Q9NSE4 | 1012 | 46.9 | 46.9 |
| I | VRS | P26640 | 1264 | 71.4 | 55.7 |
| I | VRS | Q5ST30 | 1063 | 53.6 | 55.7 |
| I | RRS | P54136 | 660 | 66.7 | 67.9 |
| I | RRS | Q5T160 | 578 | 39.2 | 39.0 |
| I | CRS | P49589 | 748 | 63.9 | 64.5 |
| I | CRS | Q9HA77 | 564 | 51.1 | 49.0 |
| I | WRS | P23381 | 471 | 70.5 | 70.3 |
| I | WRS | Q9UGM6 | 360 | 54.3 | 53.5 |
| I | MRS | P56192 | 900 | 68.8 | 68.3 |
| I | MRS | Q96GW9 | 593 | 53.3 | 50.4 |
| I | YRS | P54577 | 528 | 76.3 | 76 |
| I | YRS | Q9Y2Z4 | 477 | 56.1 | 53.4 |
| I | ERS | Q5JPH6 | 523 | 56.0 | 56.2 |
| I | QRS | P47897 | 775 | 72.3 | 72.8 |
| II | ARS | P49588 | 968 | 67.4 | 67.4 |
| II | ARS | Q5JTZ9 | 985 | 39.5 | 39.3 |
| II | GRS | P41250 | 739 | 63.5 | 64.4 |
| II | FRS-*alpha* | Q9Y285 | 508 | 67.4 | 68.4 |
| II | FRS-*alpha* (M) | O95363 | 451 | 53.1 | 51.8 |
| II | FRS-*beta* | Q9NSD9 | 589 | 60.8 | 61.7 |
| II | HRS | P12081 | 509 | 66.7 | 62.3 |
| II | HRS | P49590 | 506 | 55.6 | 62.2 |
| II | KRS | Q15046 | 597 | 75.1 | 73.5 |
| II | PRS | Q7L3T8 | 475 | 48.4 | 49.8 |
| II | NRS | O43776 | 548 | 81.7 | 80.6 |
| II | NRS | Q96I59 | 477 | 44.4 | 44.3 |
| II | DRS | P14868 | 501 | 75.5 | 74.9 |
| II | DRS | Q6PI48 | 645 | 49.5 | 41.2 |
| II | SRS | P49591 | 514 | 81.7 | 81.7 |
| II | SRS | Q9NP81 | 518 | 56.9 | 53.8 |
| II | TRS | P26639 | 723 | 79.7 | 80.2 |

**
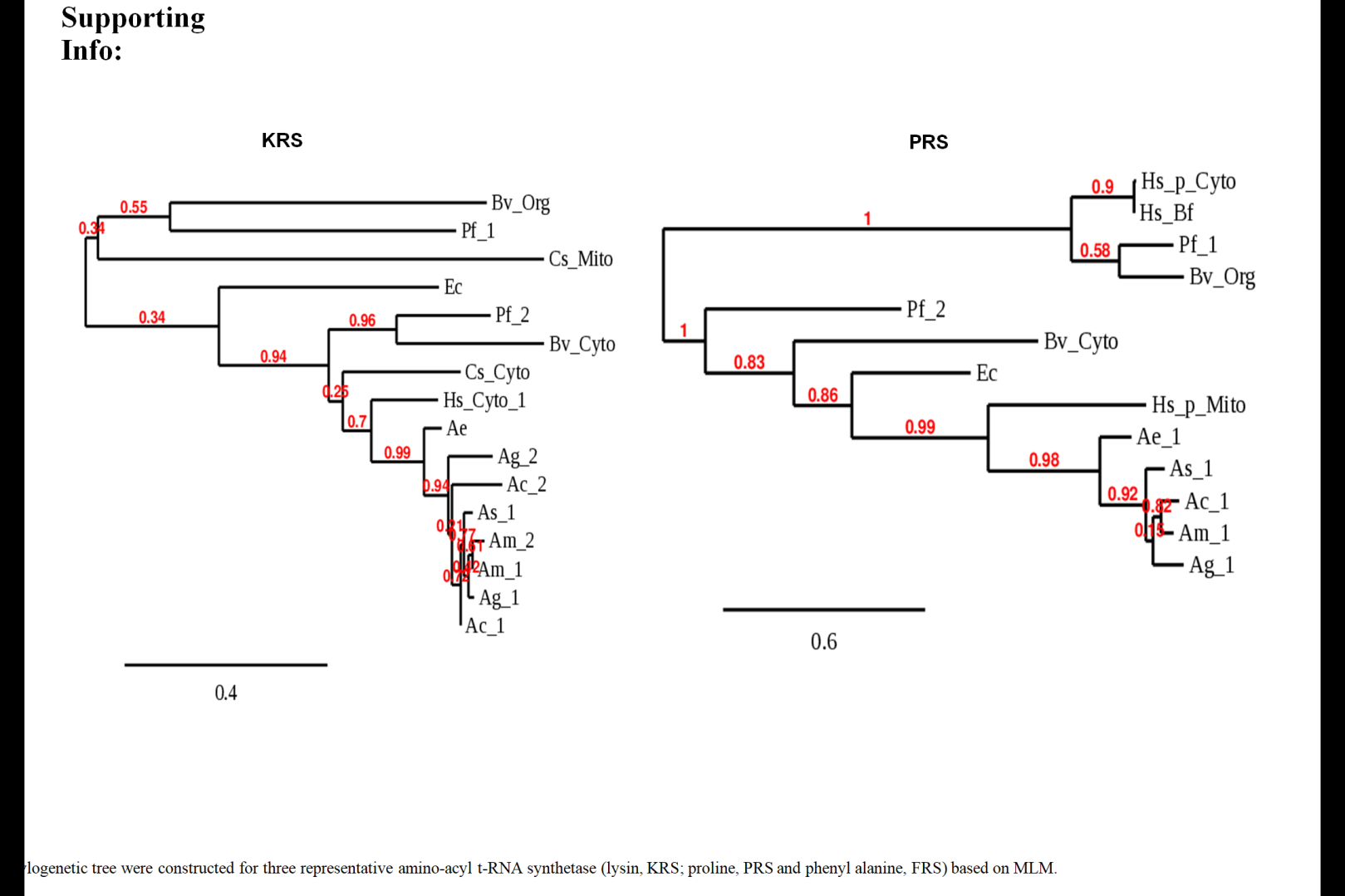
**

**A.**

**
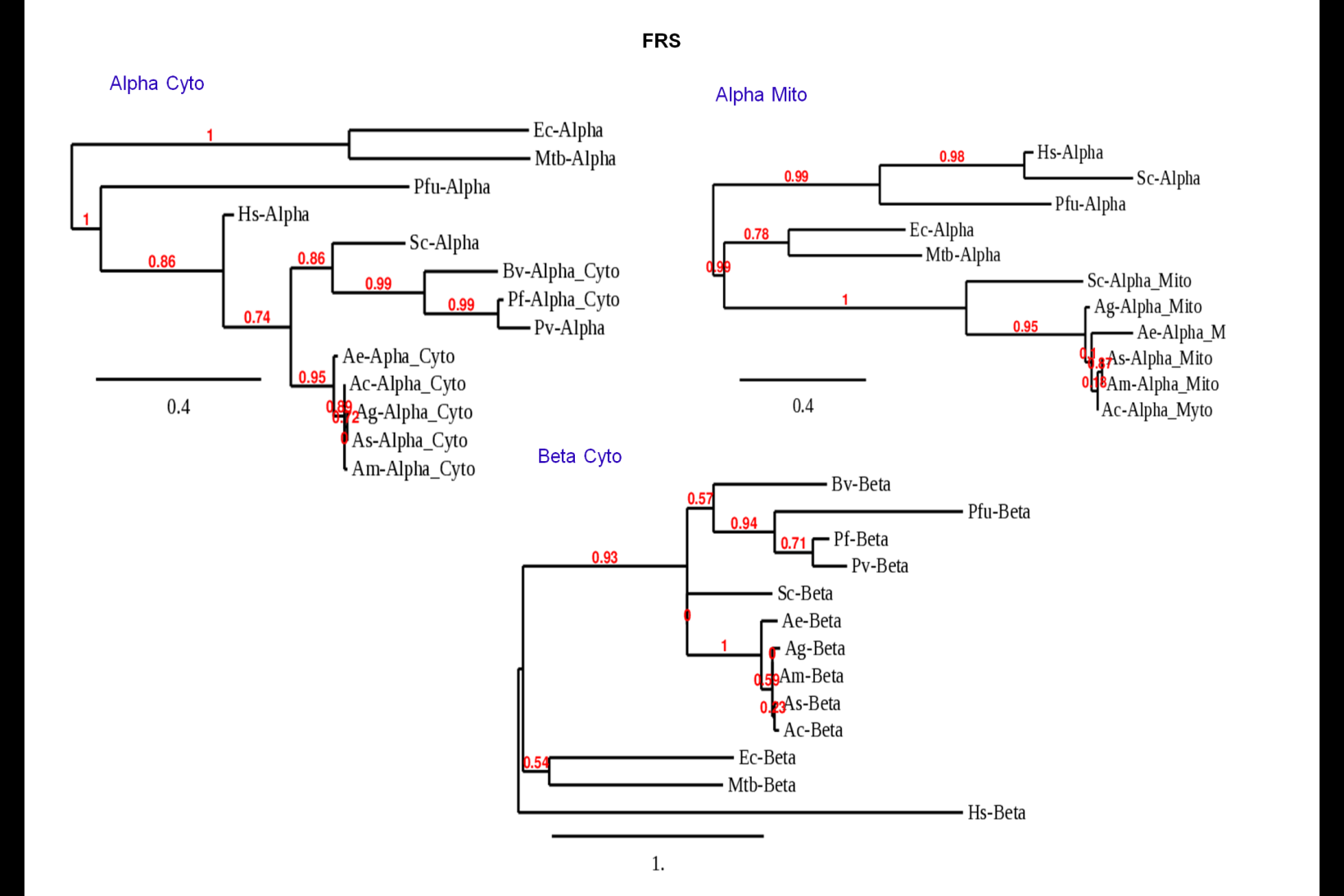
Figure S1.** Phylogenetic tree were constructed for three representative aminoacyl t-RNA synthetases (lysyl-, KRS; prolyl-, PRS and phenylalanyl-, FRS) A. For lysyl and prolyl aminoacyl t-RNA synthetases phylogenetic tree were generated using maximum likelihood method (MLM). B. In case of FRS both alpha and beta subunit was considered for phylogeny analysis. *Sc: Saccharomyces cerevisiae*; *Pf*: *Plasmodium* *falciparum*; *Hs*: *Homo sapiens*; *Ec*: *Escherichia* *coli*; *Bv*: *Babesia* *bovis*; *Ae*: *Aedes* *aegyptie*; *Ag*: *Anopheles* *gambiae*; *Ac*: *Anopheles* *culicifacies*; *As*: *Anopheles* *stephensii*; *Am*: *Anopheles* *minimus*. *Mtb: Mycobacterium tuberculosis*. *Pfu: Pyrococcus furiosus*.

**B.**
